# Statistical Analysis of Optimal Ultrasound Emulsification Parameters in Thistle-Oil Nanoemulsions

**DOI:** 10.1007/s11743-016-1887-7

**Published:** 2016-10-06

**Authors:** Małgorzata A. Miastkowska, Marcin Banach, Jolanta Pulit-Prociak, Elżbieta S. Sikora, Agata Głogowska, Michał Zielina

**Affiliations:** 1 Faculty of Chemical Engineering and Technology, Institute of Organic Chemistry and Technology, Cracow University of Technology, Warszawska 24, 31-155 Cracow, Poland; 2 Faculty of Chemical Engineering and Technology, Institute of Chemistry and Inorganic Technology, Cracow University of Technology, Warszawska 24, 31-155 Cracow, Poland; 3 Faculty of Environmental Engineering, Institute of Water Supply and Environmental Protection, Cracow University of Technology, Warszawska 24, 31-155 Cracow, Poland

**Keywords:** Experimental design, Nanoemulsions, Statistical analysis, Thistle oil, Ultrasounds

## Abstract

Thistle oil (INCI: *Silybum marianum* seed oil) is known as an anti-oxidant, moisturizing and skin regenerating cosmetic raw material. Nanoemulsions are a new form of cosmetic product showing very good user properties (ease of spreading over the skin with no greasy feeling). Moreover, due to their structure, they can also transport both hydrophilic and hydrophobic active substances to the skin. The aim of this work was the preparation and characterization of nanoemulsions, based on thistle oil. The non-ionic surfactants polysorbate 80 (PEG-20 sorbitan monooleate), decyl glucoside, and a polyglyceryl-4 ester blend were applied to stabilize the nanosystems. All formulations were obtained by a high energy method, using an ultrasonic device (Labsonic U, an ultrasound homogenizer). Variations in the emulsification parameters were tested, including surfactants concentration, pre-emulsification time, ultrasound power and sonication time. On the basis of statistical analysis (experimental design, cluster analysis, classification and regression trees) the best emulsification process parameters were determined. In order to verify the results of statistical analysis, once more an experimental study was conducted. The results obtained confirmed that statistical analysis can be a useful method in determining the conditions for obtaining stable nanoemulsions with desired properties. Formulations obtained with the use of Silybum marianum seed oil were characterized by long-term stability, a low polydispersity index, low viscosity and an average droplet size less than 200 nm.

## Introduction

Thistle oil (*Silybum marianum* seed oil) is known as a rich source of silymarin, and flavonolignans such as silybin, isosilybin, silydianin and silychristin. The substances have effects on keratinocyte proliferation and the production of extracellular matrix proteins such as type I collagen, elastin and laminin. Topical treatments containing silymarin reduce wrinkles in human skin [1–4]. Apart from regenerating and moisturizing properties, the oil also shows antioxidant activity and thanks to that it has been used in formulations intended for problem skin conditions.

Nanoemulsions are isotropic, kinetically stable dispersions of oil and water, stabilized by an interfacial film of surfactant molecules [5, 6]. They show some advantages compared to classic emulsions. Due to the very small droplet size (20–500 nm) nanoemulsions penetrate into the skin relatively easily with increased bioavailability. These preparations have been the focus of many research teams because of their wide range of applications in the pharmaceutical, cosmetic and chemical industries [7–12].

Nanoemulsions may be prepared by low- and high-energy emulsification methods. The low-energy emulsification methods (PIC, PIT) rely on chemical energy stored in surfactants. Mechanical energy is used to produce nanoemulsions using ultrasound, high-pressure homogenization and high-shear homogenization [13–15]. Applying ultrasound enhances the energy production efficiency. The ultrasound homogenization process is widely used because of the preparation of high-quality nanoemulsions (uniform droplet size, low polydispersity index) [12, 16, 17]. Moreover, ultrasound processes are known to be facile and cost-reducing [13]. Ultrasonic emulsification is based on two mechanisms: breaking the disperse phase into a continuous phase via acoustic waves, followed by acoustic cavitation, which results in the formation of micro-bubbles into smaller droplets under reduced pressure [12, 16].

Compared to the microfluidization process, the ultrasound method is more economical and practical in terms of scale-up production costs [18]. Production of nanoemulsions on a larger scale has been already investigated. In studies of Parthasarathy *et al.* [19] obtaining of palm oil-based O/W submicron-emulsions of curcumin has been performed in a liquid whistle hydrodynamic cavitation reactor. The average size of the particles obtained was around 500 nm.

As the literature reveals, statistical analysis is a useful tool in the development of nanoemulsion preparation by the ultrasound method. It allows minimization of the number of experimental trials [13, 18].

The aim of this work was to study the influence of emulsification parameters (surfactant concentration, pre-emulsification time, ultrasound power and sonication time) on the physicochemical properties of the *Silybum marianum* seed oil-based-nanoemulsions, such as average droplet diameter, size distribution, viscosity and appearance of the nanoemulsions obtained. Statistical analysis was applied in order to select the best process parameters and the quantitative composition of the nanoemulsions.

## Experimental Procedure

### Materials

The nonionic surfactants polysorbate 80 (HLB = 15.0), polyglyceryl-4 ester blend (INCI: polyglyceryl-4 laurate/sebacate, polyglyceryl-4 caprylate/caprate and water; HLB = 15.0, trade name Natragem S150) and decyl glucoside (HLB = 12.8, trade name Plantacare 2000UP) were purchased from Caesar & Lorentz GmbH, Croda Poland and BASF, respectively. The oil phase, thistle oil (INCI: *Silybum marianum* seed oil), was obtained from Olvita. Deionized and Milli-Q filtered water was used as the aqueous phase of the emulsions.

### Nanoemulsion Preparation

Nanoemulsions consisted of thistle oil, surfactant and deionized water. The concentration of oil in the emulsion systems was held constant at 10 wt%. The surfactant concentration was varied and set at three different levels (Table 1). All formulations were prepared in two stages. In the course of pre-emulsification, the crude emulsions were obtained using a magnetic stirrer (IKA^®^ C-MAG HS 7). Pre-emulsification was carried out for various pre-emulsification time periods and with different stirring speeds. After pre-emulsification, ultrasonic emulsification was performed using a Labsonic U ultrasonic homogenizer (B. Braun Biotech International GmbH) with an operating frequency of 20 kHz. The device consists of a generator, a transducer and a metal probe. The tip horn was placed in the pre-emulsified sample, and then the ultrasonication process was carried out at various ultrasonic power levels with a constant emulsification time (5 min). Table 1 presents the variability ranges of the parameters that were set during the emulsification processes. For all emulsifiers, the variability ranges of the parameters were the same.

**Table 1:** The variability ranges of emulsification independent parameters

Emulsifier	Emulsifier concentration (%)	Pre-emulsification		Ultrasonic emulsification
		Rotation of magnetic stirrer (rpm)	Stirring time (min)	Ultrasound power (%)
Polysorbate 80	2, 4 or 6	100, 300 or 500	5, 10 or 15	20, 40 or 60
Decyl glucoside				
Polyglyceryl-4 ester blend				

### Nanoemulsion Characterization

#### Droplet Size Determination

The average internal phase droplet size of the emulsions was measured by the dynamic light scattering (DLS) method, using a Malvern Zetasizer Nano ZS device. In order to avoid multiple scattering effects, samples were diluted to 1 wt% with deionized water before the measurement. The emulsion particle size was estimated by the average of three measurements and presented as the arithmetic average of the droplet diameter. DLS analysis also provided the polydispersity index (PDI) as well as a multi-modality (*N*) value as a function of the particle fraction intensity* n*(*I*) and volume* n*(*V*). These parameters give information on the droplet size distribution.

#### Stability Studies

The stability of emulsions was evaluated by measuring the droplet size by the DLS method, for fresh samples and after 60 days of storage at a constant temperature (25 °C).

#### Rheology Analysis

In order to determine the rheological properties of the obtained formulations, a Brookfield rotational R/S Plus rheometer with a C75-1 cone-plate measuring system was used. The applied cone type enables measuring viscosity in the range of 10 mPa·s to 10 kPa·s. Measurements were carried out at a constant temperature (25 °C) which was provided by a Huber Ministat 125. The viscosity values reported were the arithmetic average of three measurements.

## Calculation

### Experimental Design

Statistical analysis techniques were used in order to design experiments and to analyze the results so that their interpretation was reliable. The analyses were conducted with STATISTICA (version 10) from StatSoft^®^, which is a universal statistical software package. The type of surfactant, surfactant concentration, speed of stirring, pre-emulsification time and ultrasound power output were independent variables. The group of dependent variables included: average droplet size, polydispersity index, multi-modality, viscosity, and sample appearance after 2 weeks. Table 2 presents the specific values of each process parameter as well as the analytical results.

**Table 2:** Matrix of the experimental design and experimental data obtained for the dependent variables

Run	Independent variables					Dependent variables					
	Type of surfactant, Em-r^**a**^	Emulsifier concentration, Em-c (%)	Speed of stirring, rpm	Pre-emulsification time, premix time (min)	Output power of ultrasound, *M* (%)	Droplet size, *d* (nm) (mean ± SD)	Polydispersity index (PDI)	Multi-modality, *N*	Viscosity for 50 1/s, *η* (mPa·s) (mean ± SD)	Visual observation (VO)	Visual observation (VO) (appearance after 2 weeks)
1	1	6	100	15	60	1819 ± 69	0.24	2	682 ± 71	Creaming	Phase separation
2	1	2	500	15	60	811.6 ± 25	0.368	2	670 ± 42	Phase separation	Phase separation
3	−1	6	500	15	20	152.1 ± 3	0.25	1	480 ± 49	Phase separation	Homogeneous
4	1	6	500	5	20	1304 ± 24	0.256	1	688 ± 11	Phase separation	Phase separation
5	1	6	100	5	20	1050 ± 21	0.219	2	740 ± 10	Phase separation	Phase separation
6	1	2	100	5	60	656 ± 16	0.236	2	620 ± 30	Phase separation	Phase separation
7	−1	2	100	15	20	226.8 ± 3	0.279	2	610 ± 27	Creaming	Creaming
8	−1	2	500	5	60	306 ± 3	0.503	3	523 ± 67	Homogeneous	Creaming
9	−1	6	100	15	60	186 ± 1	0.151	1	514 ± 66	Homogeneous	Homogeneous
10	1	2	500	15	20	572.2 ± 4	0.134	1	721 ± 4	Homogeneous	Phase separation
11	−1	6	500	5	60	169.1 ± 15	0.386	3	577 ± 11	Creaming	Creaming
12	−1	2	100	5	20	203.7 ± 2	0.243	1	469 ± 23	Creaming	Creaming
13	−1	4	300	10	40	209.1 ± 1	0.285	1	517 ± 33	Creaming	Homogeneous
14	1	4	300	10	40	939.9 ± 9	0.205	1	692 ± 2	Phase separation	Phase separation
15	0	2	300	10	40	713.6 ± 20	0.478	3	756 ± 86	Creaming	Phase separation
16	0	6	300	10	40	592.8 ± 46	0.595	3	500 ± 73	Homogeneous	Phase separation
17	0	4	100	10	40	157.3 ± 1	0.142	1	733 ± 57	Creaming	Phase separation
18	0	4	500	10	40	1442 ± 48	0.918	3	656 ± 5	Creaming	Creaming
19	0	4	300	5	40	248 ± 4	0.279	1	653 ± 100	Creaming	Creaming
20	0	4	300	15	40	1054 ± 30	0.796	3	694 ± 74	Homogeneous	Phase separation
21	0	4	300	10	20	205 ± 1	0.218	1	626 ± 28	Phase separation	Homogeneous
22	0	4	300	10	60	4005 ± 103	0.357	2	699 ± 26	Homogeneous	Phase separation
23	0	4	300	10	40	146.8 ± 2	0.395	2	597 ± 14	Homogeneous	Homogeneous
24	0	4	300	10	40	150.6 ± 5	0.282	2	510 ± 44	Homogeneous	Phase separation
25	0	4	300	10	40	154.5 ± 3	0.169	2	540 ± 20	Creaming	Homogeneous

^a^Em–r: −*1* polysorbate 80, *0* decyl glucoside, *1* polyglyceryl-4 ester blend

### Cluster Analysis and Classification and Regression Trees

Table 2 shows the parameters for which the physicochemical characterization was made. Based on the variables (average particle size, polydispersity index, modality, viscosity and appearance) characterizing the studied objects (nanoemulsion compositions), they were divided into characteristic groups using cluster analysis. The basic idea is to divide the similar objects into groups of objects which are similar to each other and which are dissimilar to objects from other groups.

The purpose of performing cluster analysis and classification and regression trees was to determine whether the resulting clusters indicate some regularities occurring in the data set. The discovery of these regularities allows for a qualitative assessment of the influence of process parameters on the properties of the prepared nanoemulsions.

Conducting cluster analysis also allows one to determine the possibility of reducing the multidimensional dataset. Thus, it is possible to reduce the number of parameters that significantly affect the studied system by means of grouping.

Analysis of agglomeration was applied first. This resulted in a cluster tree (dendrogram) which shows the structure of objects due to declining similarity between them. As a rule of binding, which specifies that two objects are sufficiently similar that they can be combined, a weighted pair-group method using arithmetic averages (WPGMA) was employed. The squared Euclidean distance was used as a measure of the distance between objects (as a function of the lack of similarity). This method was used to determine the number of clusters and their number was confirmed in k-means analysis. On this basis, clusters differing from each other as much as possible were determined. Variables were standardized just before performing the analysis.

In order to indicate the independent variables which had the greatest influence on the measured parameters, the ranking of predictors validity was done.

Due to the existence of independent and dependent variables having qualitative and quantitative characteristics, classification and regression trees were applied to build predictive and descriptive models. Classification trees are used in modeling processes when the dependent variable is expressed on a nominal or ordinal scale. Regression trees are used when the variable is measured on at least an interval scale. The aim of building a predictive model is to provide a qualitative or quantitative prediction of the phenomenon, while the aim of building a descriptive model is the description and presentation of patterns in the studied population. The purpose of building of a model is to obtain homogeneous subclusters from the dependent variable point of view.

The classification and regression trees (C&RT) algorithm from the module of interactive trees was used in the analysis.

## Results and Discussion

### Statistical Analysis Results

#### Cluster Analysis

On the basis of the data presented in the Experimental design section (Table 2), the prepared nanoemulsions may be divided into groups, and this division may be explained in a logical manner. Moreover, it is possible to make a qualitative interpretation of the influence of process parameters on the physicochemical properties of the products and to choose the best parameter values. The highest quality products were characterized by a small average droplet size, low polydispersity index, low viscosity and long-term stability. These kinds of output parameters were the most favorable ones.

**Fig. 1:**
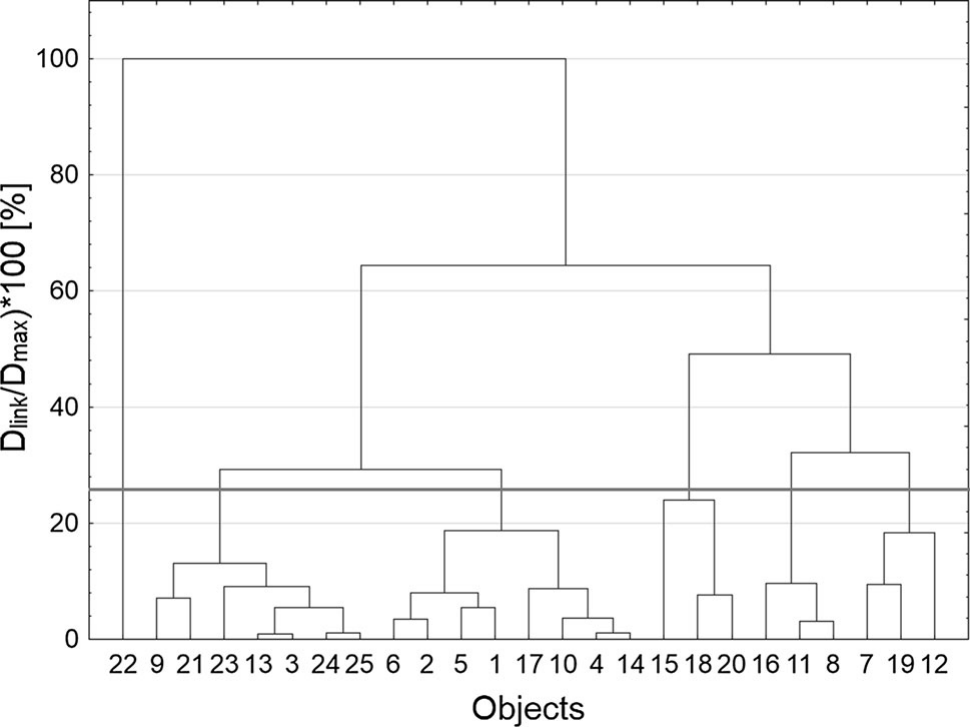
Tree diagram classifying the objects

The hierarchical tree shown in Fig. 1 is the most important analysis result to be considered. The plot in Fig. 1 was prepared using a standardized scale of the vertical axis. Six clusters of objects were obtained. Object no. 22 was characterized by an exceptionally large size of the particles, which means that it is an unusual observation and it creates a one-item cluster. Cluster 2 consisting of objects no. 3, 9, 13, 21, 23, 24 and 25 was characterized by a small particle size, low and moderate values of the polydispersity index and low and moderate viscosity. The distribution of nanoparticle size was mono- or bimodal. All products classified into the Cluster 2 were homogeneous. Cluster 3 consisted of objects no. 1, 2, 4, 5, 6, 10, 14 and 17. They were characterized by a moderate or large particle size, average values of PDI and high viscosity. These were nanoemulsions that delaminated immediately after preparation. Object no. 17 is an exception, as it was characterized by small nanoparticles, but after 14 days it delaminated as well. Clusters 4 and 5 consisted of objects no. 8, 11, 16 and 15, 18, 20, respectively. They were characterized by a small/moderate or moderate/large particle size, moderate or high PDI and moderate or high viscosity. The droplet size distribution was trimodal. These products creamed immediately after preparation. Moreover, after 14 days, objects no. 15, 16 and 20 delaminated. Cluster 6, comprising objects no. 7, 12 and 19, was characterized by a small particle size, moderate values of the polydispersity index and low/moderate viscosity. Polymodality and moderate values of PDI were confirmed by creaming of the emulsions obtained.

By weakening the criteria of object similarity, it may be concluded that cluster 6 aggregates with cluster 5, creating a new cluster that can be aggregated with cluster 4. Thus, it can be concluded that the objects assigned to these three clusters showed the greatest mutual similarity, determined primarily by the creaming of samples.

**Fig. 2:**
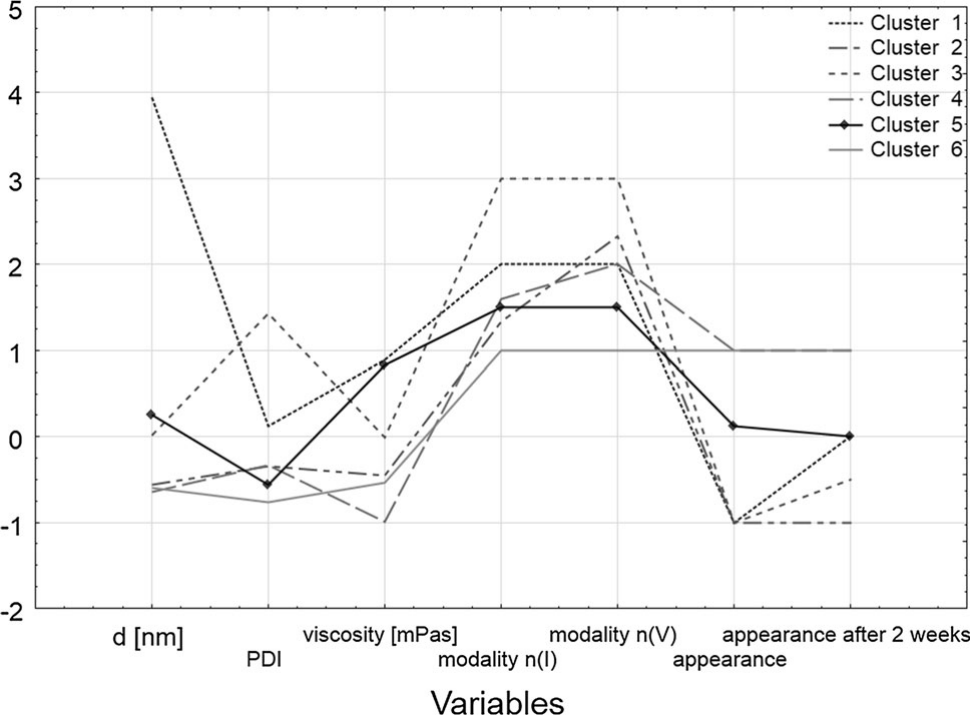
The k-means plot

In order to confirm these conclusions, clustering by the k-means method was applied. Figure 2 shows a line graph of means for each cluster, which is a graphical comparison of the differences between them.

Table 3 lists the elements of each cluster.

**Table 3:** Elements of each cluster

Cluster number					
1	2	3	4	5	6
Objects					
22	2, 12, 19, 7	20, 8, 18, 16, 15, 11	25, 3, 13, 23, 24	14, 1, 2, 5, 6, 4, 10, 17	21, 9

Cluster 4 includes homogeneous objects which had low viscosity, but did not have a monomodal nanoparticle size distribution. Two-elemental cluster 6 was the most desirable one because of the physical and chemical parameters of the objects obtained. Objects 9 and 21 were homogeneous and characterized by a small particle size, a low polydispersity index, low viscosity and a monomodal particle size distribution. Based on these results, it can be concluded that the most favorable process parameters for preparing nanoemulsions were as follows: when using polysorbate 80 at a concentration of 6 %, mixing should be carried out at a speed of 100 rpm, the premix time should be 15 min and the output power of ultrasound should reach 60 %; when using decyl glucoside at a concentration of 4 %, mixing should be carried out at a speed of 300 rpm, with a premix time of 10 min and the power should reach 20 %. An increase in the rotation speed in the case of the process based on polysorbate 80 makes the products lose their monomodal particle size distribution, and thus they are classified into cluster 4. This cluster also contains objects which were obtained using decyl glucoside, but using an ultrasound power equal to 40 %.

Clusters 2 and 3 consist of objects that creamed. They were also characterized by a polymodal droplet size distribution. Elements of cluster 3 are characterized by trimodality. A reduction in ultrasound power (down to 20 %) or an increase in stirrer speed (up to 500 rpm) in the processes in which polysorbate 80 was used caused creaming in the products obtained. Creaming also occurred in the products obtained using decyl glucoside. This process was influenced by the emulsifier concentration and premix time. An increase in ultrasound power (up to 60 %) also caused both creaming and an increase in particle size. Based on the analysis of the results presented in Fig. 2 and in Table 3, one can confirm the earlier thesis that cluster 1 contains objects characterized by a different particle size, i.e. about 4000 nm.

Cluster 5 consisted of objects that delaminated. These samples were obtained with polyglyceryl-4 ester blend, although one product was based on decyl glucoside. Delamination of the product that was emulsified with decyl glucoside was due to a too low stirrer speed.

#### Analysis of Variance

Table 4 presents the results of variance analysis, confirming the importance of variables which were the basis for object grouping (*p* < *α* = 0.05). The results confirm that the variable “appearance” was the main criterion for grouping objects into clusters. Appearance was also an essential quality parameter of the prepared nanoemulsions.

**Table 4:** Analysis of variance

	Between SS	*df*	Within SS	*df*	Test *F*	Significant *p* value
Droplet size, *d*	19.8793	5	4.1207	19	18.3320	0.0000
Polydispersity index, PDI	16.7715	5	7.2285	19	8.8167	0.0002
Viscosity, *η*	12.4465	5	11.5535	19	4.0937	0.0108
Modality,* n*(*I*) as the intensity function	11.4933	5	3.8667	19	11.2952	0.0000
Multi-modality,* n*(*V*) as the volume function	10.2933	5	2.6667	19	14.6680	0.0000
Appearance	16.9650	5	0.8750	19	73.6766	0.0000
Appearance after 2 weeks	11.4600	5	1.5000	19	29.0320	0.0000

#### Classification and Regression Trees

**Fig. 3:**
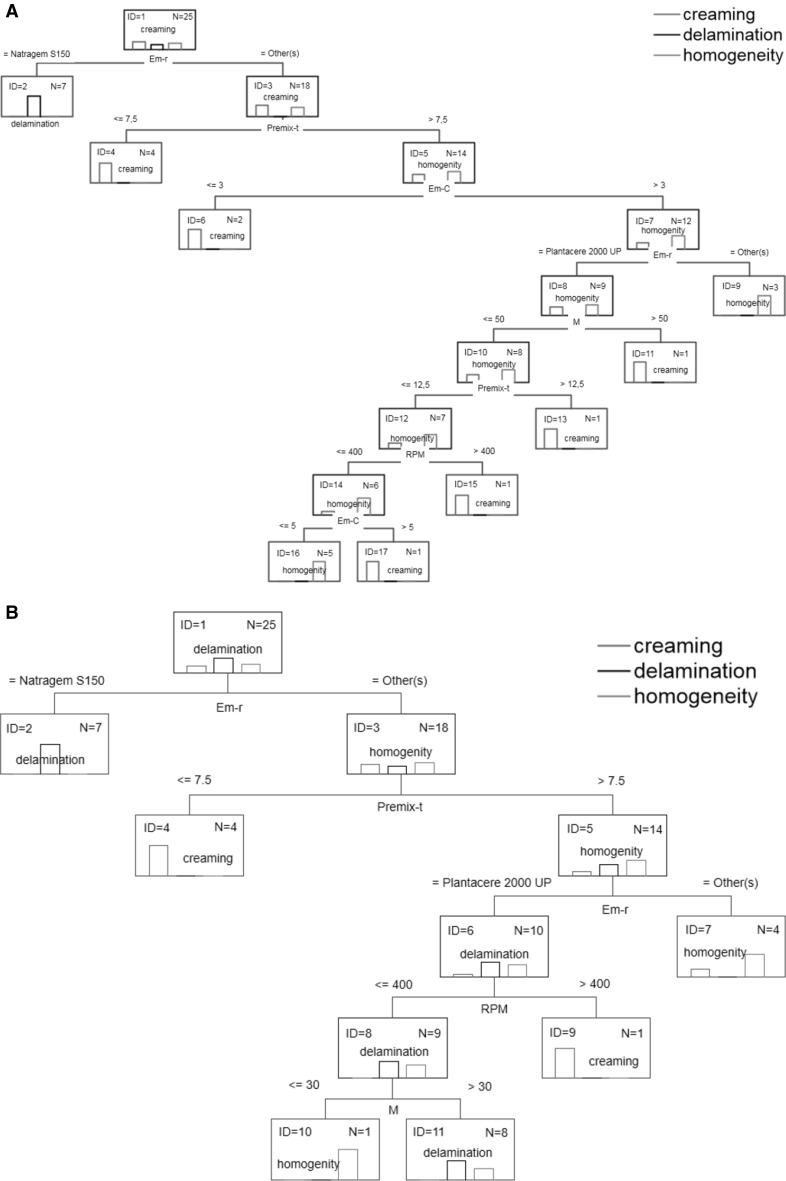

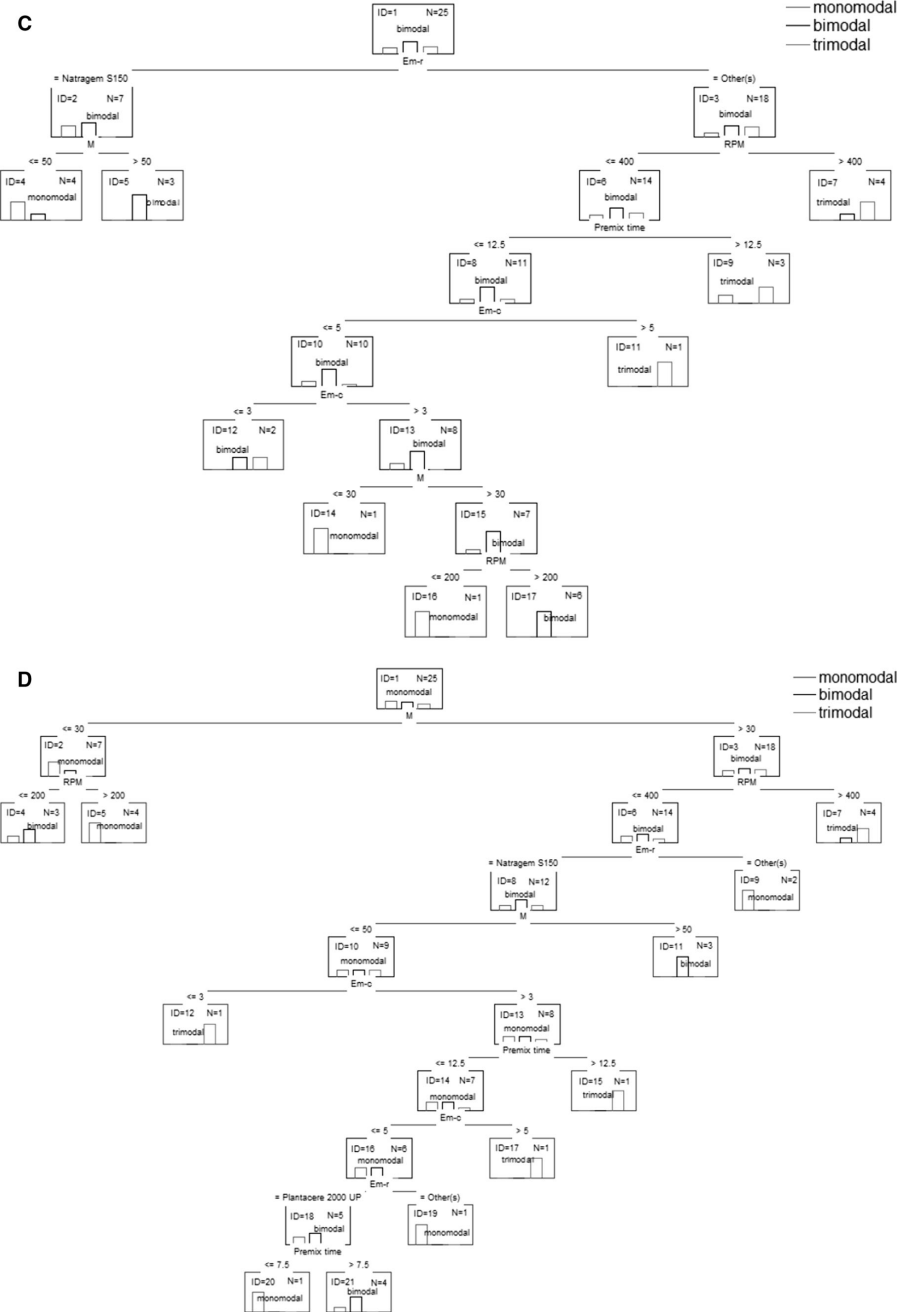

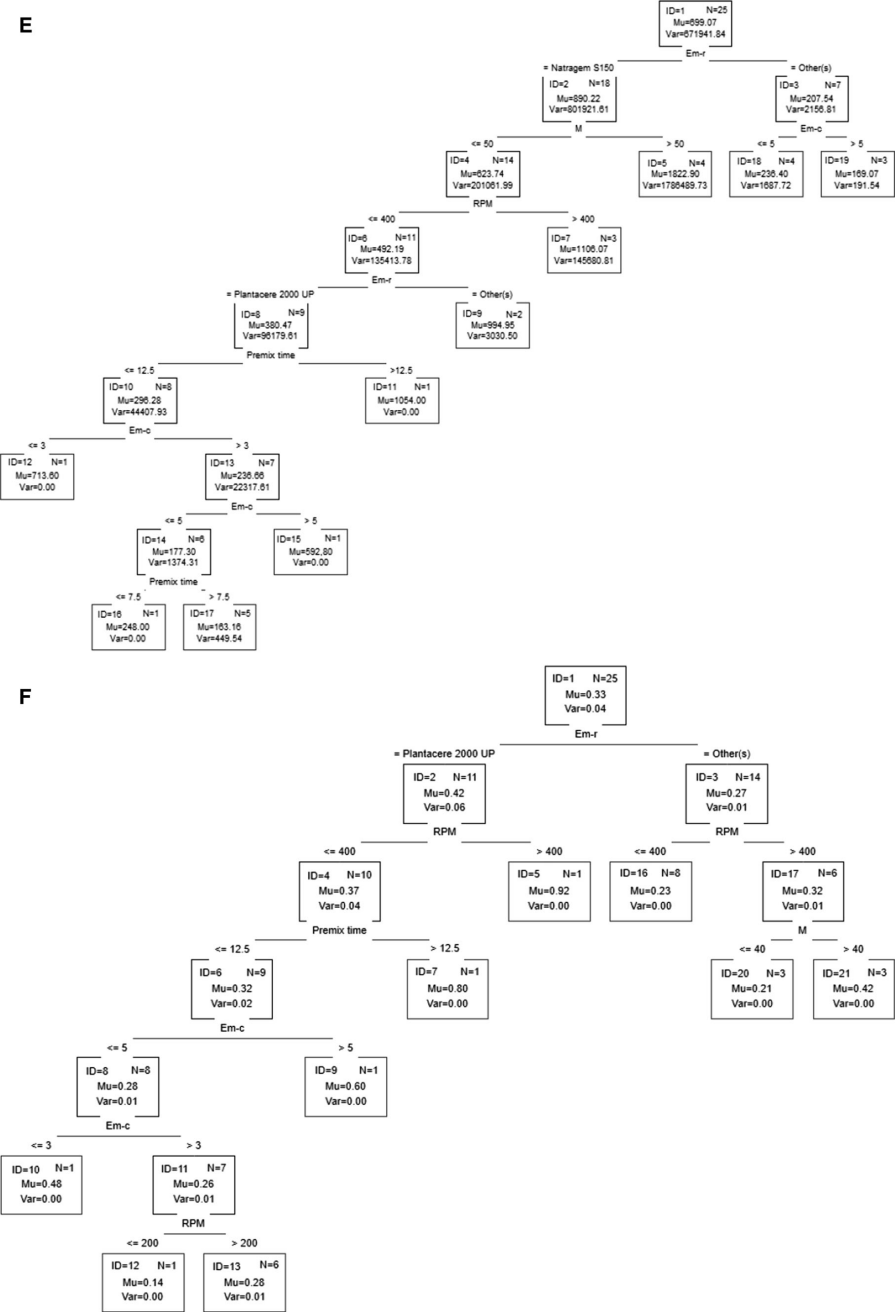

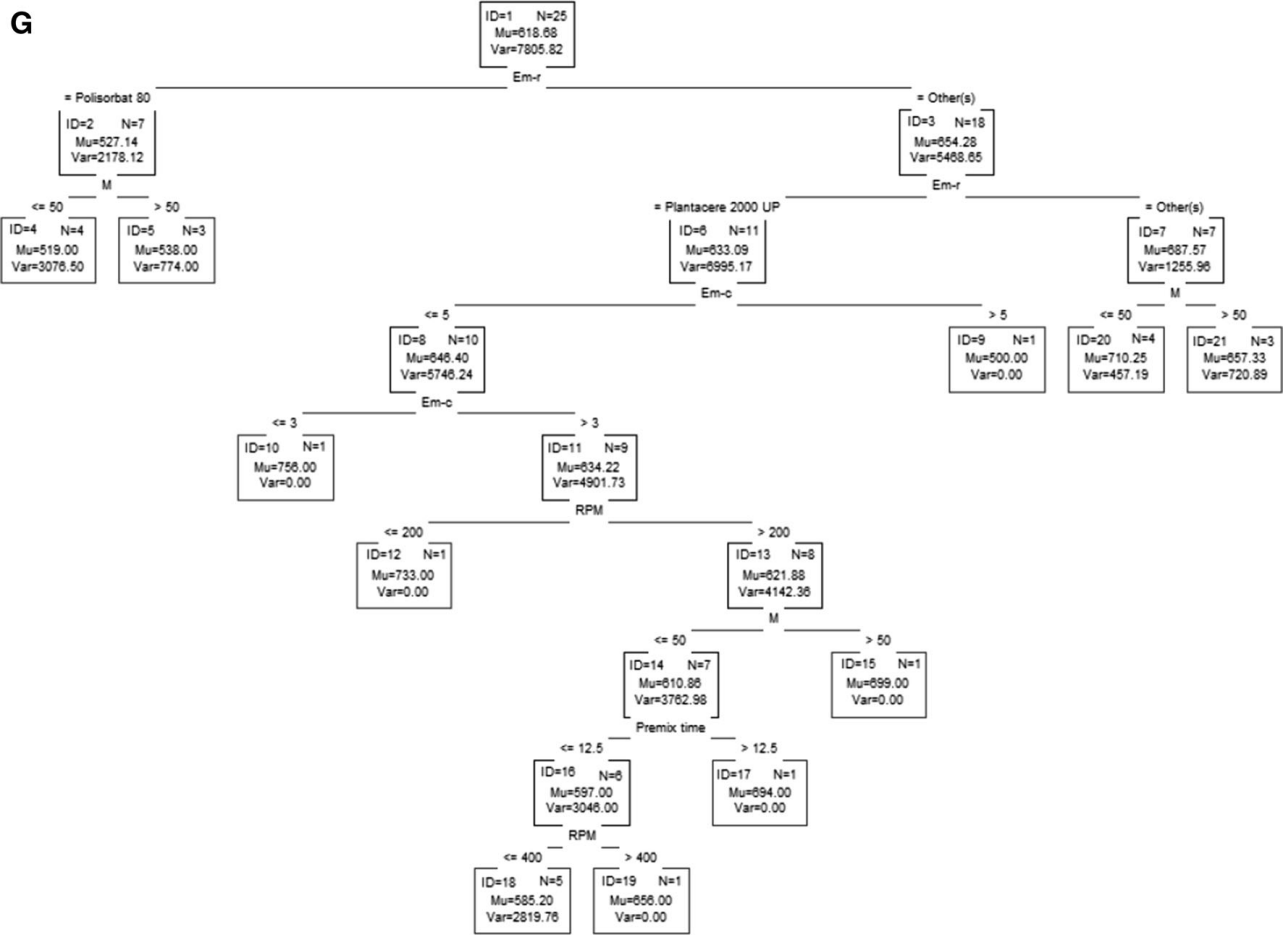
Classification and regression trees **a** appearance, **b** appearance after 2 weeks, **c** *n*(*V*), **d** *n*(*I*), **e** droplet size, **f** polydispersity index, **g** viscosity

Classification and regression trees are a partitioning method. As a result, classification and regression trees for predicting continuous dependent variables (regression) and categorical predictor variables (classification) are built. The trees presented in Fig. 3 show the graphical results of analysis.

On the basis of the classification tree shown in Fig. 3a, one can conclude that uniform products may be obtained using polysorbate 80 or decyl glucoside as the emulsifier. Moreover, polysorbate 80 should be used at a concentration greater than 3 %, and the process should be performed with a premix time >7.5 min. These conditions ensure homogeneity of the product after 2 weeks of aging (Fig. 3b). In order to obtain a homogeneous product when using decyl glucoside as the emulsifier, its concentration should be in the range of 3–5 %, the stirrer speed should be less than 400 rpm, the premix time should be between 7.5 and 12.5 min and the ultrasound power should be set at 50 % (Fig. 3a). Moreover, if the ultrasound power is reduced to a value less than 30 %, a product that maintains homogeneity over 14 days will be obtained (Fig. 3b).

The product will be characterized by monomodality n(V) if polysorbate 80 or decyl glucoside is used as the emulsifier and if the stirrer speed is equal to or less than 400 rpm, the premix time is equal to or less than 12.5 min, the emulsifier concentration is greater than 3.5 % and the power is equal to or less than 30 %. A similar effect may be achieved after changing the stirrer speed to 200 rpm or less and setting the ultrasound power above 30 %. Furthermore, if the stirrer speed is in the range of 200–400 rpm and polysorbate 80 is used as the emulsifier, products characterized by monomodality n(I) will be obtained. In the case of using decyl glucoside, an additional condition must be met: the premix time should be equal to or less than 7.5 min.

One can obtain a product based on a polyglyceryl-4 ester blend which would be characterized by monomodality n(I), but such a product does not comply with the other quality parameters.

Taking into account the ranges of the process parameters, it can be concluded that in order to obtain homogenous and monomodal (both V and I) products, the process must be carried out with the use of polysorbate 80 or decyl glucoside as the emulsifying agent. If polysorbate 80 is used at a concentration greater than 3 %, the stirrer speed is equal to or less than 400 rpm, the premix time is greater than 7.5 min and the ultrasound power is greater than 30 %, or if decyl glucoside is used at a concentration in the range of 3–5 %, the stirrer speed is equal to or less than 400 rpm, the premix time is greater than 7.5 min and the power is greater than 30 %, the resulting products will be characterized by the most favorable parameters defined by a set of dependent variables of a qualitative nature.

For the specific values or variation ranges of the process parameters, on the basis of regression trees (Fig. 3 e–g), one can make a prediction of the average particle size, PDI and the viscosity of the nanoemulsions obtained. When polysorbate 80 is used as an emulsifier and the process conditions required for obtaining the best quality parameters are fulfilled, the nanoemulsion will be characterized by a particle size in the range of 163–236 nm, the polydispersity index will be equal to about 0.232 and the viscosity will be around 527 mPa·s. In the case of using decyl glucoside, the particle size is expected to be in the range of 169–236 nm, the PDI will be equal to about 0.258 and the viscosity is expected to be around 585–733 mPa·s.

#### Ranking of Predictors Validity

**Fig. 4:**
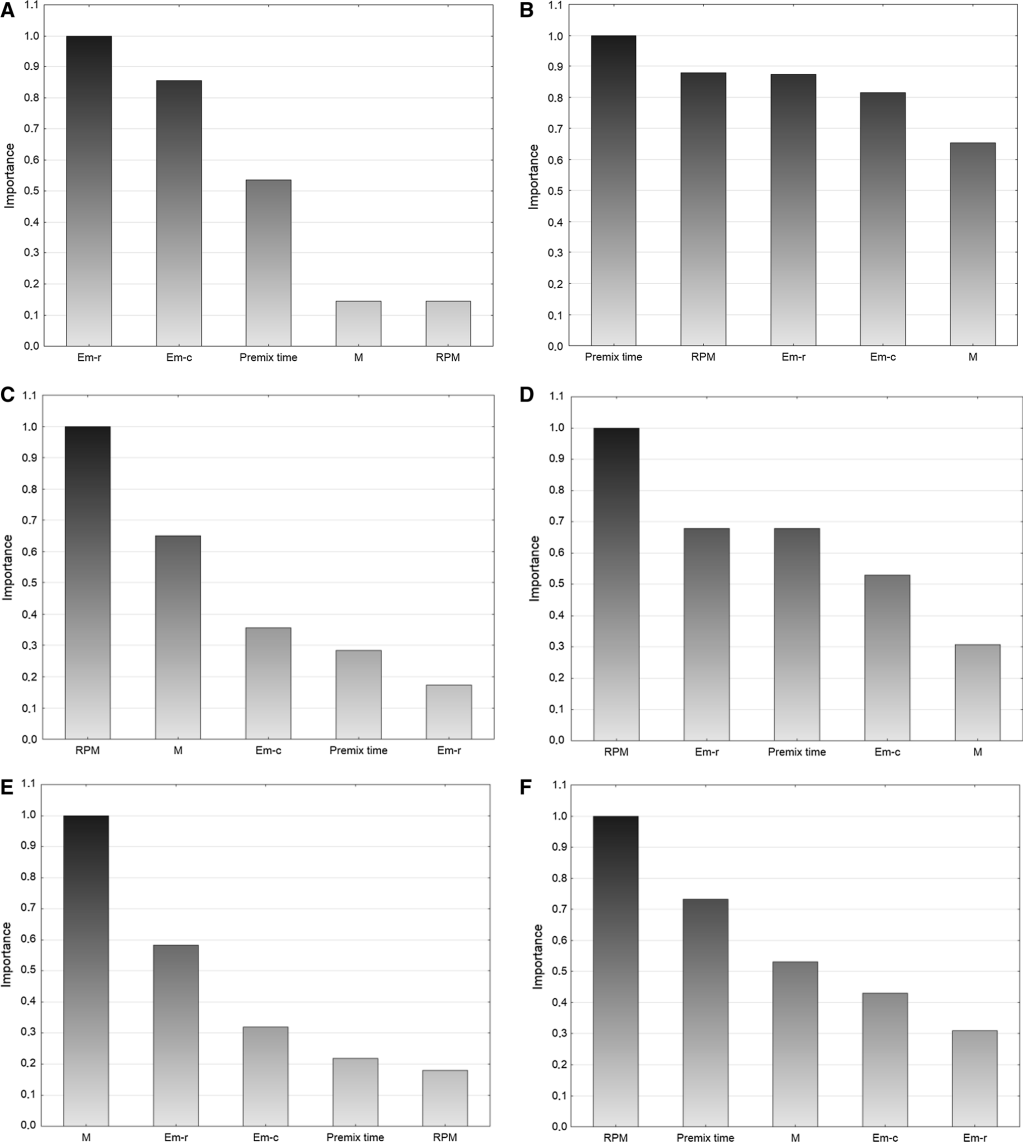

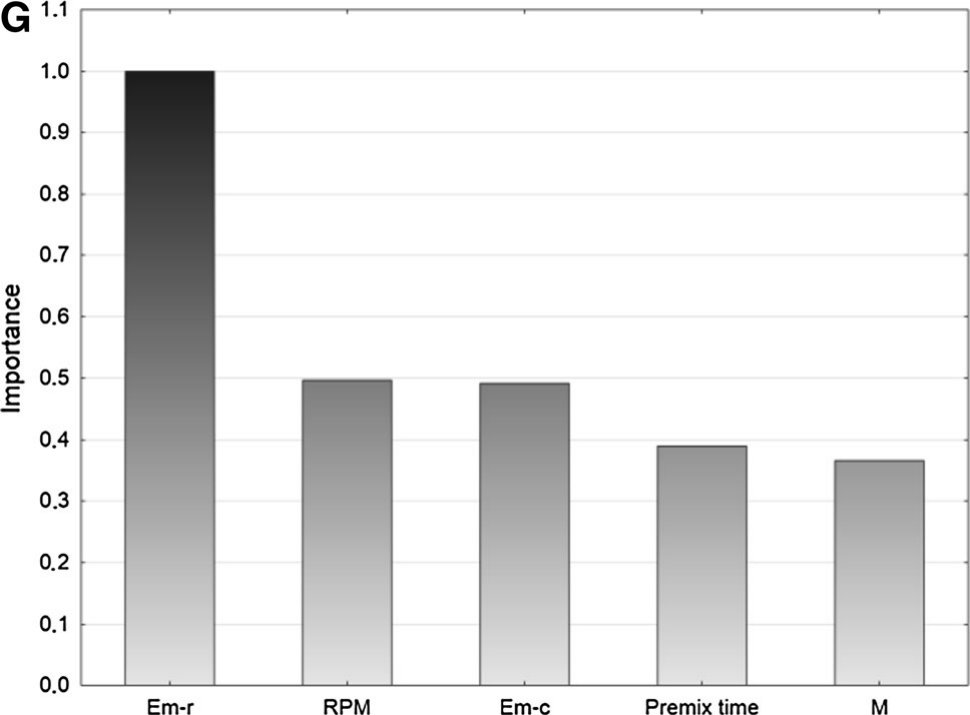
Importance plots **a** appearance, **b** appearance after 2 weeks, **c** *n*(*V*), **d** *n*(*I*), **e** droplet size, **f** polydispersity index, **g** viscosity

The priority of predictors (Fig. 4) presents the independent variables that are ordered on a scale from 0.0 to 1.0, where the highest values represent the greatest impact of each variable on the dependent variables.

The appearance of products is mostly determined by the type of emulsifier and its concentration. The premix time also affects the appearance of the nanoemulsion. The premix time plays a role as a factor determining the appearance of products after 2 weeks. The speed of stirring and the emulsifier type affect the stability of emulsions in a slightly weaker manner. The* n*(*V*) is mostly dependent on the speed of stirring, ultrasound power and emulsifier concentration. The modality is influenced by the speed of stirring and to a similar degree by the type of emulsifier and the premix time. Ultrasound power has the highest impact on droplet size. It is also affected by the type and concentration of the emulsifier. Polydispersity is mainly dependent on the speed of stirring, the premix time and ultrasound power. The type of emulsifier and its concentration as well as the speed of stirring have the highest impact on product viscosity.

#### Verification of the Models

In order to verify the regression trees obtained, additional tests were carried out. Table 5 presents the specific parameters which were set in order to conduct the verification analysis. The results are also shown in Table 5. It also presents the properties (droplet size, polydispersity index) of nanoemulsions after 60 days of storage at room temperature (25 °C). Based on these results, it may be concluded that the applied models describe the process very well. One may notice the high consistency of the results. The data obtained in the course of these experiments (26–29) fit the value ranges which were predicted during C&RT analysis. This means that the model has been properly fitted and one may rely upon it.

**Table 5:** The best emulsification process parameters obtained based on regression trees with measured and observed physicochemical properties of the obtained nanoemulsions

Run	Independent variables					Dependent variables						
						*T* = 1 day					*t* = 60 days	
	Type of surfactant, Em-r^a^	Emulsifier concentration, Em-c (%)	Speed of stirring, rpm	Pre-emulsification time, premix time (min)	Output power of ultrasounds, M (%)	Droplet size,* d* (nm) (mean ± SD)	Polydispersity index (PDI)	Multi-modality, n(I)	Viscosity for 50 1/s, *η* (mPa·s) (mean ± SD)	Visual observation (VO)	Droplet size,* d* (nm) (mean ± SD)	Polydispersity index (PDI)
26	0	4	100	10	20	189.0 ± 5	0.216	1	580 ± 50	Homogeneous	233 ± 12	0.325
27	0	4	300	10	20	179.8 ± 10	0.303	1	562 ± 30	Homogeneous	168 ± 9	0.232
28	−1	4	300	15	60	191.1 ± 9	0.335	1	690 ± 100	Homogeneous	201 ± 3	0.269
29	−1	6	100	10	40	159.5 ± 8	0.257	2	587 ± 22	Homogeneous	164 ± 6	0.295

^a^Em-r: −*1* polysorbate 80, *0* decyl glucoside

As shown, the properties of the nanoemulsions such as the average droplet size and polydispersity index are almost unchanged after a long period of time. These results indicate that the applied models provide kinetically stable nanoemulsions.

When it comes to the surfactant type, the results obtained show that the properties of the nanoemulsions stabilized by synthetic (polysorbate 80, HLB = 15.0) and natural emulsifier (decyl glucoside, HLB = 12.7) are comparable. However, in the case of decyl glucoside with the lower HLB value (around 12–13), the minimum particle size (186 nm) and the lowest polydispersity index (0.151) were obtained (sample 25, Table 2). Medina *et al.* also noted that better properties were achieved with nanoemulsions based on a natural oil (soya oil) and stabilized by soybean lecithin or Poloxamer 188, a natural emulsifier [20]. Other research groups [19, 21] noticed that the minimum droplet size and maximum emulsion stability were obtained for O/W emulsions stabilized by surfactant with an HLB value in the range from 10 to 12.

However, it was noted that increasing the polysorbate 80 concentration (up to 6 %) led to a reduction in droplet size, because sufficient surfactant reduces the interfacial tension, stabilizes the system and prevents the coalescence of droplets. In the case of emulsions stabilized by decyl glucoside, the best results were noticed at the 4 % concentration of this surfactant. Further addition of surfactant (up to 6 %) did not induce a reduction in droplet size because the concentration of emulsifier in the bulk sample allowed rapid diffusion and adsorption of the surfactant to newly formed droplets. Too high a surfactant concentration may result in a lower diffusion rate of surfactants and may cause the coalescence of emulsion droplets. This observation agrees with the literature [13, 21].

The results show that, in most samples, the minimum particle size was obtained when the ultrasound power was at an intermediate level (40 %). A higher ultrasound intensity caused an increase in the particle diameter. Jafari *et al.* [22] called this phenomenon "over processing". Increasing the intensity of ultrasound power could drive emulsion droplets to the nodes and antinodes of the acoustic field. The closer proximity of droplets may result in droplet coalescence. This observation is in agreement with the results of other research groups [17, 21]. Li and Chiang [17] and Kentish *et al.* [23] hypothesized that this phenomenon could be due to the fact that, at a higher applied power, increasement of droplet coalescence takes place. Also, Tang *et al.* [18] observed that increased intensity of emulsification might increase droplet deformation and the disruption of emulsion droplets. It is likely that applying a higher acoustic amplitude increases the energy input, which may disrupt the emulsifier interfacial layer.

Statistical methods in development of nanoemulsions have been used by Alzorqi *et al.* [24]. They applied them in studies on obtaining of palm-olein based nanoemulsions by using ultrasounds technique. The influence of independent variables such as water content, oil/surfactant ratio, ultrasonic power, irradiation time and combinations of these parameters on droplets size, polydispersity index and viscosity was studied. Authors applied central composite design. Based on response surface methodology technique it was possible to determine the strict values of independent variables that affected specific values of output parameters.

Tan *et al.* [25] applied Box–Behnken design and response surface methodology in order to study sterically stabilized nanodispersions of curcumin. It has been demonstrated that few minutes of ultrasonication allow obtaining narrow particles size distribution. Their stability is affected by HLB value of surfactants used in the formulations.

## Conclusion

For the specific values or variation ranges of selected process parameters, on the basis of regression trees, a prediction of the average particle size, PDI and the viscosity of nanoemulsions was performed. Data obtained in the course of the experiments (26–29) fit the values which were predicted during the C&RT analysis. This means that the model properly fitted and one may rely upon it. Moreover, the properties of the analyzed nanoemulsions, such as the average droplet size [*d* (nm)] and polydispersity index (PDI) were almost unchanged after 60 days of storage, which means that the obtained formulations can be characterized by long-term stability. Uniform products may be obtained using polysorbate 80 or decyl glucoside as the emulsifier. However, in the case of the natural surfactant, a lower surfactant concentration and lower ultrasound power were required to obtain nanoemulsions with the optimal physicochemical properties.

This research did not receive any specific grant from funding agencies in the public, commercial, or not-for-profit sectors.
